# A challenging diagnosis of alpha-1-antitrypsin deficiency: identification of a patient with a novel F/Null phenotype

**DOI:** 10.1186/1710-1492-7-18

**Published:** 2011-11-13

**Authors:** Michael R Ringenbach, Erin Banta, Melissa R Snyder, Timothy J Craig, Faoud T Ishmael

**Affiliations:** 1 Department of Medicine, Section of Allergy and Immunology, The Pennsylvania State University Milton S. Hershey Medical Center, 500 University Dr., Hershey, PA 17033, USA; 2 Department of Biochemistry and Molecular Biology, The Pennsylvania State University College of Medicine, 500 University Dr., Hershey, PA 17033, USA; 3 Department of Laboratory Medicine and Pathology, Mayo Clinic College of Medicine, Hilton 2-10D, 200 First St. SW, Rochester, MN, 55905, USA

**Keywords:** Alpha-1-antitrypsin, diagnostic testing, phenotype, genotype

## Abstract

Alpha-1-antitrypsin (A1AT) deficiency is a genetic disease characterized by low levels and/or function of A1AT protein. A1AT deficiency can result in the development of COPD, liver disease, and certain skin conditions. The disease can be diagnosed by demonstrating a low level of A1AT protein and genotype screening for S and Z mutations, which are the most common. However, there are many genetic variants in A1AT deficiency, and this screening may miss rarer cases, such as those caused by dysfunctional protein. We identified a patient with a previously unreported F/null phenotype that was missed by routine screening. This case highlights the wide variation in possible mutations, limitations in diagnostics, and the importance of combining clinical suspicion with measurement of protein levels, phenotypic analysis, and in appropriate cases expanded genetic analysis.

## Background

Alpha-1 Antitrypsin (A1AT) is a serine protease inhibitor that is encoded by the SERPINA 1 gene located on chromosome 14 [1,2]. It is a highly effective inhibitor of neutrophil elastase, and serves a protective function to prevent excessive proteolysis of matrix components in the airways and other organs [1,2]. A wide variety of mutations in the SERPINA1 gene are possible, and these can result in low/absent levels or non-functional protein [1-3]. A1AT deficiency results in early onset emphysema, and may induce liver disease, necrotizing panniculitis, or a C-ANCA positive vasculitis [1,2].

A1AT deficiency is most often seen in a population of European origin, but can affect all races [3]. It is estimated that in the United States there is 1 case per 3000-5000 persons, though the carrier frequency of mutant alleles has been reported to be 2-3% of the American population, suggesting that the prevalence may be higher than this [1-3]. It is also estimated that only about 10% of the patients with this condition are currently identified, due in part to low recognition of the disease, genetic heterogeneity, and complexities of diagnosis [1-3].

The normal A1AT gene product is designated Pi*M, and there more than 100 known variants [1]. The most common mutant alleles are Pi*Z and Pi*S, and inheritance of these as homozygous alleles (i.e. Pi*ZZ, Pi*SS) or compound heterozygotes (Pi*SZ) results in a deficiency state [1-3]. These mutations are characterized by low levels and dysfunction of A1AT. Rare null mutations result in completely absent protein, and others, such as F, confer dysfunctional protein with normal levels [4-6]. Available diagnostic tests include measurement of protein levels, genotyping for common mutations, or phenotypic analysis of variants by isoelectric focusing (IEF) [7]. However, given the wide spectrum of mutations, diagnosis can be challenging and use of any of these tests alone can be misleading.

We present an A1AT-deficient patient with a novel and previously undescribed F/null phenotype who was initially misdiagnosed based on protein level and genotyping. This case highlights the genetic heterogeneity of the disease and complexities in diagnosis of A1AT deficiency.

## Case Report

The patient is a 64 year old male who was seen with a chief complaint of dyspnea and recurrent skin rash. His dyspnea had been persistent and worsening in nature since he was diagnosed with COPD at age 49. Treatment with fluticasone 220 mcg 2 puffs twice a day via a HFA inhaler did not provide benefit, and he required use of albuterol as a rescue inhaler multiple times per week. He had a 10 pack-year history of cigarette-smoking, but stopped 7 years ago. His rash was described as diffuse, pruritic, erythematous, and raised, consistent with urticaria. It started approximately eight months ago and presented multiple times per week. Treatment with antihistamines (cetirizine 10 mg daily, hydroxyzine 50 mg every 6 hours as needed) and prednisone 40 mg daily were both ineffective. There were no known triggers and no implicated drugs or foods. His past medical history was otherwise significant only for chronic rhinitis. He had no history of liver disease or vasculitis. Review of systems was unremarkable. There was no family history of lung disease, liver disease, or autoimmunity.

Medications at presentation included cetirizine 10 mg daily, fluticasone HFA 220 mcg, two puffs twice a day, albuterol HFA as needed, mometasone nasal spray and azelastine nasal spray.

Physical examination revealed bilateral wheezing and a generalized erythematous, maculopapular rash predominantly on the torso and arms. The remainder of the physical exam was otherwise unremarkable. He previously had numerous blood tests performed, including LFTs, CBC, Hepatitis serologies, HIV screening, TSH, Free T4, ANA CH50, ANCA and CRP, which were all within normal limits.

Pulmonary function testing revealed an FEV1 of 1.82 L (52% of predicted) with an FEV1/FVC ratio of 70%. He failed to demonstrate significant reversibility with albuterol treatment, (post-bronchodilator increase in FEV1 of 9%). He was found to have a reduced DLCO of 17.73 ml/min/mmHg (62% of predicted), and evidence of air trapping with a RV of 3.26 L (127% of predicted). The latter may have led to an artifactually low FVC resulting in an FEV1/FVC ratio that was only mildly decreased. His pulmonary function tests over the past four years were also reviewed, which showed a 24.7% decrease in FEV1 over that time period, despite treatment with inhaled corticosteroids and long acting bronchodilators.

A skin biopsy was performed on an urticarial lesion, which showed a perivascular infiltration of mostly neutrophils without any evidence of blood vessel damage, consistent with a neutrophil-predominant urticaria.

Given the poor response to treatment and the early onset of COPD with a minimal smoking history, the diagnosis of A1AT deficiency was considered. Serum testing for A1AT revealed a borderline-low level of 93 mg/dL (normal range is 100-190 mg/dL). A genotype analysis was performed at a commercial laboratory, which was reported as a normal MM genotype.

**Figure 1:**
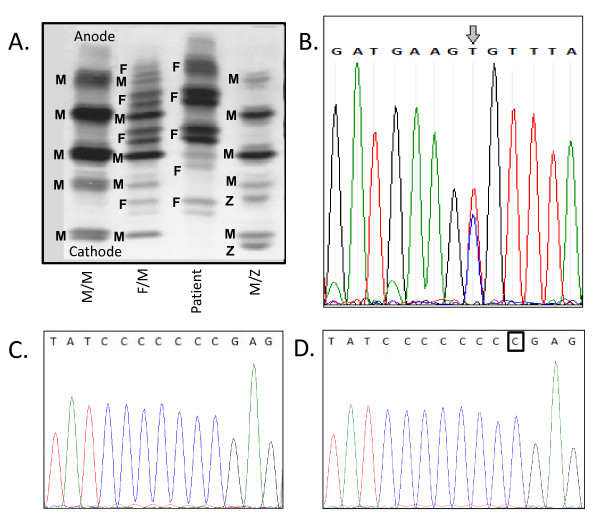
**Identification of the patient's phenotype and genotype**. ***A***, Determination of phenotype by IEF of A1AT isotypes in the patient and controls. Comparison of the patient's sample with controls of the M/M, M/F, and M/Z phenotype revealed that the patient's serum contained only F bands (M, F, Z bands indicated by labels on gel). ***B***, DNA sequencing of exon 4 demonstrated a T/C SNP at nucleotide 739 (R247C). C, sequencing of exon 5 showed the presence of a normal allele (***C***) and a C insertion at nucleotide 1158 (***D***).

Because of the clinical history of COPD and a discrepancy between the low A1AT serum levels and reported MM genotype, we remained suspicious that the patient could have a functional A1AT deficiency and performed a phenotype analysis. Isoelectric focusing (Figure 1A) indicated the presence of an F phenotype, and based on the low A1AT level, it was suspected that this represented an F/null phenotype. The F/null phenotype is extremely rare, and would be missed by the commercial genotype screen, which probes the most common mutations, S and Z. In these cases, the result may be reported by default as MM.

To confirm the patient's F genotype, we sequenced the A1AT gene in our research laboratory. As shown in Figure 1B, we demonstrated that one allele harbored a T/C single nucleotide polymorphism (SNP) at nucleotide 739 in exon 4 of the A1AT gene, resulting in an Arg to Cys change (R247C). This mutation has previously been described to confer an F phenotype, consistent with the IEF analysis [4,5]. This is a rare mutation that results in production of a dysfunctional protein, usually with normal levels.

As the 739 T/C mutation was present in only one allele and only F bands were observed on IEF, we hypothesized that the patient's phenotype was F/null and sequenced the rest of the A1AT gene. Amplification of exon 5 of the A1AT gene from genomic DNA and sequencing of the PCR-product suggested that there was a heterozygous frameshift at nucleotide 1158. To further analyze this, the PCR products were cloned into a TOPO2.1 vector and sequenced. As expected, we observed insertion of a C at nucleotide 1158 in approximately half of the samples screened and the normal sequence in the others, indicating heterozygosity (Figure 1C, D). The 1158 C insertion results in a frameshift that changes crucial amino acids in the active site at the C-terminus of the protein and introduces a premature stop codon after amino acid 399. This mutation has not been described, but is similar in nature to the rare Null Bolton mutation, which arises from a deletion of a C at the same position and also produces a frameshift with a premature stop codon [6,8].

The F mutation produces in a protein that lacks activity, such that patients with homozygous F/F phenotypes develop A1AT deficiency despite having normal protein levels [4,5]. Our patient was a compound F/null heterozygote, and thus would be expected to produce reduced levels of A1AT protein that had reduced function. Complicating the diagnosis, however, was that the A1AT protein level was only slightly decreased at 93 mg/dL, the lower limit being 100 mg/dL. Thus, clinical suspicion and further phenotype testing and gene sequencing was needed to establish the diagnosis.

This case of A1AT deficiency highlights the variability of possible mutations, and raises a number of questions related to diagnosis of the disease. A1AT deficiency should be considered in all cases of COPD, especially so in patients without a significant history of smoking, in young patients, and in patients with asthma when significant obstruction persists after treatment. In this case in particular, our patient demonstrated a 25% decrease in FEV1 over the span of four years, which increased our suspicion of the disease.

However, given the large number of potential mutations, diagnosis of A1AT deficiency can be challenging. In this patient, we had a high index of suspicion for A1AT deficiency, but a protein level was only slightly below the lower limit of normal and genotyping was reported as MM. Measurement of A1AT levels alone can be misleading. First of all, A1AT is an acute phase reactant and levels can vary from depending on many factors and from one blood draw to another [1]. Secondly, in cases such as F/F phenotypes, the A1AT level will be normal, thought the protein is dysfunctional. Furthermore, the range of normal levels of A1AT is quite broad, the lower limit may vary by lab (as low as 90 mg/dL), and even patients with low normal or normal levels by report may be deficient.

Thus, low levels of A1AT should prompt further investigation and further evaluation by phenotypic analysis. Not all laboratories utilize an algorithm to perform phenotypic analysis in cases where the A1AT level is low, so the physician ordering the tests should keep this in mind. However, even when levels are normal, if there is clinical suspicion, phenotypic analysis is warranted to diagnosis deficiency due to dysfunctional protein.

Genotype testing at most commercial labs utilize probes to the most common mutations, and combined with A1AT levels may also be used to screen for A1AT deficiency. However, this may miss rare mutations (as in our case), and it is important to note that although a genotype may be reported as MM, it may rather reflect an absence of the most common S and Z mutations. In fact, we would argue that all labs that utilize PCR to probe for these two mutations should report the results in this manner to reduce confusion. More extensive gene sequencing may be necessary in select cases to indentify rare mutations. In any case, the clinical history, A1AT level measurement, phenotype analysis, and genotyping should be used in concert, and physicians should be aware of limitations of each of the diagnostics tests.

Our patient was started on A1AT replacement therapy and has had no further decline in his lung function or worsening of symptoms, though long term monitoring of lung function is needed to determine if treatment will slow the rate of FEV1 decline. The neutrophilic urticaria was treated with plaquenil 200 mg twice a day, and symptoms have improved. It is not clear if urticaria is a unique finding related to this specific mutation, though a neutrophilic panniculitis has been described in A1AT deficiency [9]. This panniculitis tends to present similarly to cellulitis and has been described in patients with heterozygous and homozygous mutations, but not in patients with the F phenotype [10].

## Conclusions

We present a patient with A1AT whose diagnosis was delayed secondary to the presence of a rare mutation and A1AT level just below normal limits. The case emphasizes the importance of maintaining a high level of suspicion for disease in patients with COPD, and reinforces the utility of A1AT screening in these patients as this disease is under-diagnosed. This case supports an algorithm of screening that includes measuring an A1AT level, followed by genotyping and phenotypic assays. The limitations of each should be considered, and in some cases more extensive gene sequencing may be necessary to confirm the genotype.

## Informed consent

Written informed consent was obtained from the patient for publication of this Case report and any accompanying images. A copy of the written consent is available for review by the Editor-in-Chief of this journal.

## Abbreviations

A1AT: alpha-1-antitrypsin; IEF: isoelectric focusing; SNP: single nucleotide polymorphism.

## Competing interests

The authors declare that they have no competing interests. TC has a research grant with CSL-Behring.

## Authors' contributions

MR performed molecular genetics studies and drafted the manuscript, EB assisted with molecular genetics studies and aided in drafting the manuscript, MS performed the isoelectric focusing experiment and aided in drafting the manuscript, TC participated in the design of the study and aided in drafting the manuscript, FI conceived of the study, and participated in its design and coordination and helped to draft the manuscript. All authors have seen the final draft of the manuscript.
